# Rescue of ankylosing hip following open reduction and internal fixation of acetabular fracture by surgical resection of heterotopic ossification: A case report

**DOI:** 10.1016/j.ijscr.2018.10.022

**Published:** 2018-10-19

**Authors:** Kazumichi Kitayama, Yohei Kawakami, Tomoaki Fukui, Keisuke Oe, Ryosuke Kuroda, Takahiro Niikura

**Affiliations:** Department of Orthopaedic Surgery, Kobe University Graduate School of Medicine, Kobe, Japan

**Keywords:** Heterotopic ossification, Acetabular fracture, Hip ankylosis, Surgical resection

## Abstract

**Introduction:**

Heterotopic ossification is a major complication after surgical treatment of acetabular fractures. Heterotopic ossification generally involves the large joints, often limits the range of motion, and may cause ankylosis.

**Presentation of case:**

This case report describes a 59-year-old man with severe heterotopic ossification who developed an acetabular fracture and resultant hip ankylosis, which was rescued by surgical resection of the heterotopic ossification. He had accompanying head injury and multiple other fractures, which were treated conservatively. Open reduction with internal fixation of the acetabular fracture was performed through the ilioinguinal and Kocher–Langenbeck combined approach. The patient unexpectedly returned to our hospital 7.5 months after the fracture surgery. We found that his left hip joint was completely ankylosed by severe heterotopic ossification. We performed surgical resection of the heterotopic ossification through a direct lateral approach 9.5 months after the initial surgery. At the final follow-up, 5.5 years after the heterotopic ossification resection surgery, the hip function including the range of motion was satisfactory. Radiographs showed no signs of recurrence, and he could walk with no support.

**Discussion:**

The only effective treatment for established HO is surgical excision. Whether delayed or early surgical resection of heterotopic ossification is more effective remains controversial.

**Conclusion:**

We considered that waiting for a long time before surgical resection of the heterotopic ossification would lead to more disability, and early resection of the heterotopic ossification was not a contraindication despite the fact that the uptake on the bone scan was still intense.

## Highlights

• Heterotopic ossification is a major complication after surgical treatment of acetabular fractures.
• Whether delayed or early surgical resection of heterotopic ossification is more effective remains controversial.
• Early surgical resection is not necessarily contraindication.

## 1 Introduction

Formation of heterotopic bone is associated with predisposing etiologies such as neurogenic, traumatic, and genetic conditions and some surgical procedures [1]. Heterotopic ossification (HO) generally involves the large joints, often limits the range of motion, and may cause ankyloses [2]. Ankylosing joints usually require surgical resection to rescue their dysfunction. Delayed HO resection has been recommended to allow for maturation of HO and to reduce the recurrence of HO and associated surgical complications. In contrast, early resection of HO was recently shown to reduce surgical complications and enable an earlier start of rehabilitation. In the present case, we performed surgical resection of HO in a patient with hip ankylosis at a relatively early period. This treatment led to a satisfactory outcome without HO recurrence for 5 years after the resection.

This work has been reported in line with the SCARE criteria [3].

## 2 Presentation of case

**Fig. 1:**
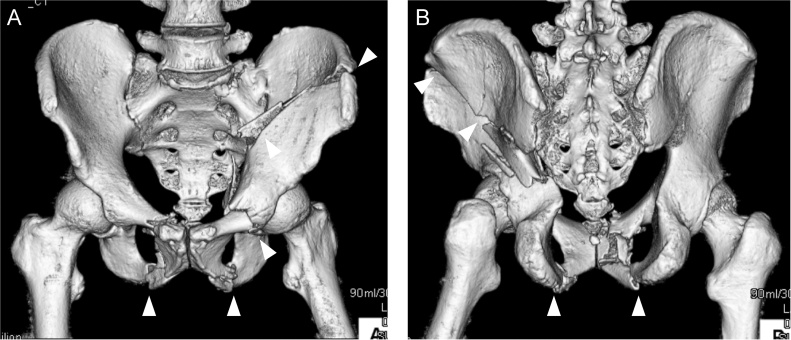
CT image of the pelvis after injury, showing the both-column fracture in the left acetabulum and bilateral pubic bone fractures (arrowheads).

A 59-year-old man was hit by a car and brought to our emergency department. His Glasgow Coma Scale score was 6 (E1V1M4) and blood pressure was low. Radiographs and computed tomography (CT) showed a both-column fracture in the left acetabulum and bilateral pubic bone fractures (Fig. 1A,B). Brain CT initially showed no signs of brain injury. We diagnosed hemorrhagic shock following the pelvic fractures and immediately performed transcatheter arterial embolization. He had accompanying injuries including an incomplete fracture of the C2 lamina, a right clavicle fracture, right rib fractures from the second to eighth ribs, and fractures of the left thumb and index finger, all of which were treated conservatively. Six days after the injury, we performed open reduction and internal fixation (ORIF) for the acetabular fracture using a low-profile pelvic plate system (DePuy Synthes Trauma, West Chester, PA, USA) through an ilioinguinal and Kocher–Langenbeck combined approach. He remained unconscious for 3 weeks, and a second brain CT scan showed a chronic subdural hematoma. He was transferred to a rehabilitation unit 1.5 months after the operation, still with a consciousness disorder.

**Fig. 2:**
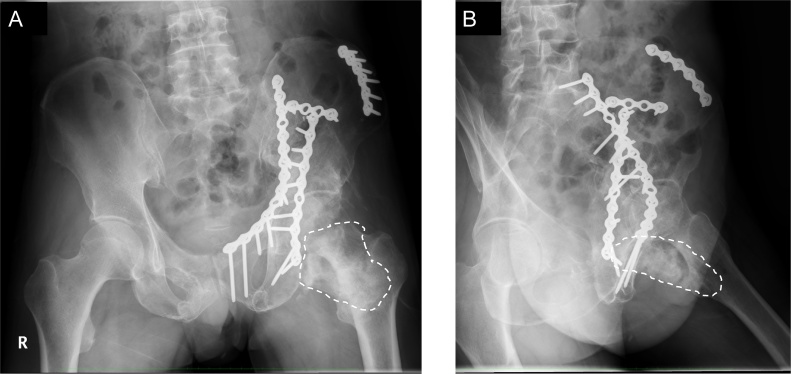
(A) Anteroposterior and (B) lateral pelvic radiographs before HO resection. HO (white dotted line) bridged the left hip posteriorly.

**Fig. 3:**
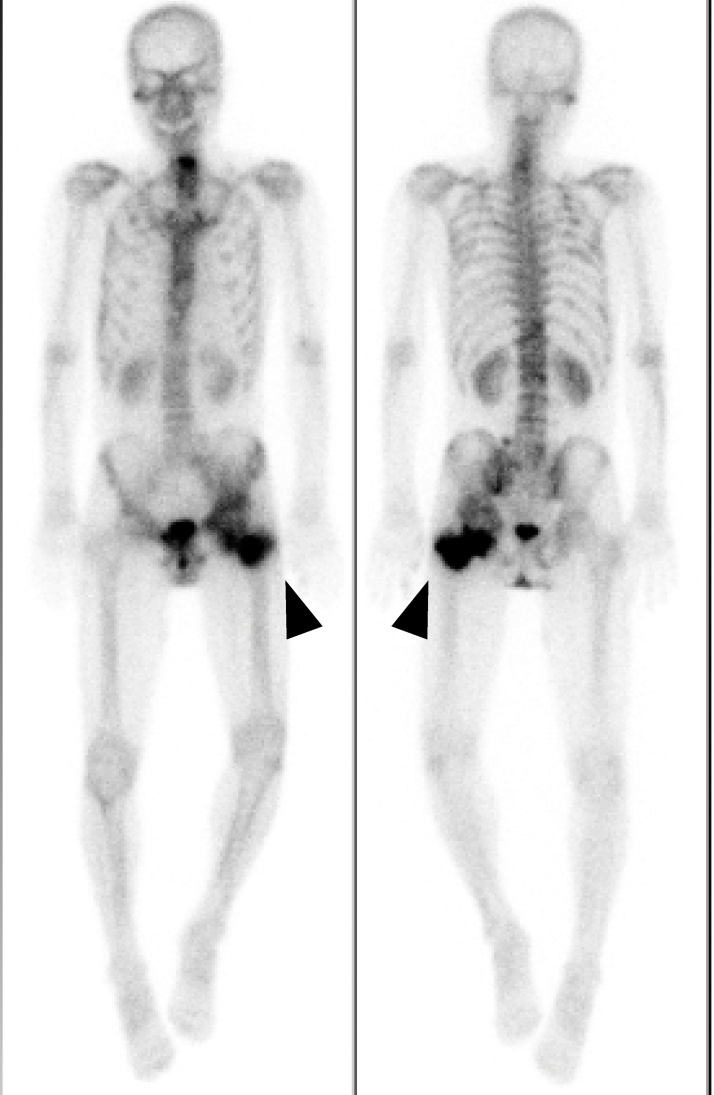
On the bone scan image, the arrowheads show increased uptake around the left hip.

The patient unexpectedly presented to our hospital 6 months after the transfer. He could walk on his own and was completely conscious and alert, but he complained of discomfort in his left hip and difficulty in its motion. Physical examination revealed that his left hip joint was completely ankylosed with a range of motion 45° in flexion, 10° in abduction, and 20° in external rotation. Radiographs and CT revealed HO (Brooker class 4) [3] around the left hip joint (Fig. 2A,B). A bone scan showed intense uptake around the left hip (Fig. 3).

**Fig. 4:**
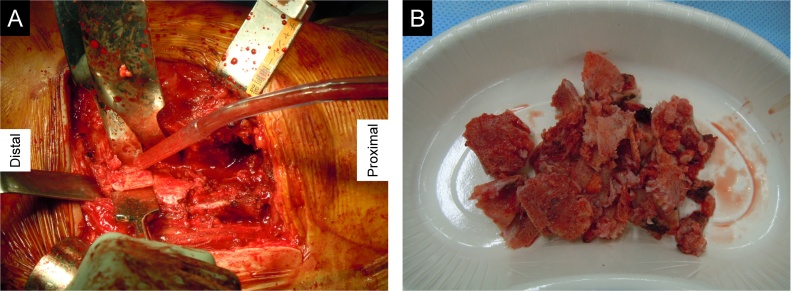
(A) Resection of HO posterior to the trochanter by a chisel through a direct lateral approach. (B) Piece-by-piece resection of HO.

We performed surgical resection of the HO through a direct lateral approach 9.5 months after the initial surgery. During the operation, we found HO posterior to the greater trochanter and identified the sciatic nerve posterior to the HO (Fig. 4). After the resection, the left hip joint regained mobility. We administered indomethacin at 75 mg/day for 6 weeks after the surgery to prevent recurrence of HO.

**Fig. 5:**
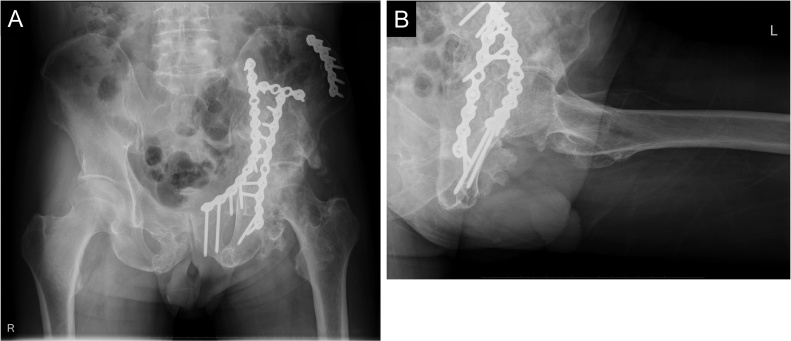
(A) Anteroposterior and (B) lateral pelvic radiographs at the final follow-up, 5.5 years after HO resection. The patient developed no HO recurrence.

At the final follow-up, 5.5 years after the HO resection surgery, the range of motion was 110° in flexion, 0° in extension, 30° in abduction, 10° in adduction, 50° in external rotation, and 0° in internal rotation. Radiographs showed no signs of HO recurrence (Fig. 5A, B), and he could walk with no support. The Japanese Orthopaedic Association hip score for his left hip was 42 of 100 before the HO resection and reached 87 of 100 after the resection.

## 3 Discussion

HO is a major complication after the surgical treatment of acetabular fractures. Giannoudis et al. [5] reported that after ORIF of acetabular fractures, the overall incidence of HO was 25.6% and the incidence of severe HO (Brooker grade 3 or 4) [4] was 5.7%. HO generally affects large joints, often limits the range of motion, and may cause ankylosis. The hip joint is one of the most frequently affected joints [6]. Several risk factors for HO formation have been reported, including severe trauma, head injury, invasive surgery, delayed surgery, prolonged intubation, and male sex [7]. In our case, the patient had multiple risk factors: severe acetabular fractures associated with head injury, performance of ORIF through the anterior and posterior combined approach, and male sex.

Nonsteroidal anti-inflammatory drugs and irradiation are used as prophylaxis against HO formation, but whether these treatments are effective remains controversial. Burd et al. [7] reported that indomethacin and irradiation are both effective prophylaxes against HO formation following surgical treatment of acetabular fractures. Kan et al. [8] found that nonsteroidal anti-inflammatory drugs could reduce the incidence of HO after total hip arthroplasty. In contrast, Griffin et al. [9] examined the effectiveness of indomethacin to prevent HO after acetabular surgery and found no reduction in the HO rate. We considered the use of indomethacin for our patient with multiple risk factors for HO formation to reduce the risk of recurrence.

The only effective treatment for established HO is surgical excision [6]. Surgical resection of HO often results in significant improvement of joint motion, but HO resection and hip release after ORIF of an acetabular fracture is a difficult procedure [10]. Many complications of hip HO resection have been reported, such as femoral neck fracture, avascular necrosis of the femoral head, sciatic nerve injury, infection, and HO recurrence [4,11,12]. Wu et al. [10] reported that the rate of HO recurrence after surgical resection of HO following ORIF of acetabular fractures was 33.3%. Genet et al. [6] reported that HO resection in an ankylosed hip was associated with more complications and less improvement in joint motion than HO resection in a non-ankylosed hip.

Previous studies have indicated that HO resection should be delayed until the HO has matured and the bone scan has normalized to reduce the risk of HO recurrence [6]. Garland and Orwin [11] performed hip HO resection in patients with spinal cord injuries, with a mean time from injury to surgery of 50.6 months. Meiners et al. [12] performed hip HO resection in patients with spinal cord injury at an average of 82.1 months after injury. Conversely, Genet et al. [6] reported that early resection of immature HO did not lead to a higher rate of recurrence. Wu et al. [10] reported no significant difference in the HO recurrence rate between early and delayed HO resection. They also reported that it was easier to identify the border between HO and normal cortex during the operation if the time interval before HO resection was short. They recommended early HO resection because it enables an easier operation, an earlier start of rehabilitation, and prevention of complications such as intraoperative fracture of the femoral neck, wound infection, and sciatic nerve injury [6,11]. Melamed et al. [13] suggested that increased uptake on bone scans was not a contraindication to surgical excision of HO, provided the neurologic status is stabilized. We performed HO resection 9.5 months after the initial operation despite the fact that the uptake on the bone scan was still intense. Cobb et al. [14] performed HO resection at a mean of 13 months after total hip arthroplasty. We considered that waiting for several years would lead to more disability in our patient, and 9.5 months after the initial operation was not a contraindication for HO resection surgery.

## Conflict of interest statement

None.

## Funding

None.

## Ethical approval

Ethical approval of a case report has been exempted by our institution.

## Consent

Written informed consent was obtained from the patient for publication of this case report and accompanying images. A copy of written consent is available for review by the Editor-in-Chief of this journal on request.

## Author contribution

Kazumichi Kitayama wrote the manuscript, acquired data, reviewed literatures.

Yohei Kawakami supervised the manuscript, reviewed literatures.

Tomoaki Fukui revised the manuscript critically for intellectual content.

Keisuke Oe revised the manuscript critically for intellectual content.

Ryosuke Kuroda revised the manuscript critically for intellectual content.

Takahiro Niikura designed and supervised the manuscript, acquired data, participated in the operation.

All authors read and approved the final version of this manuscript.

## Registration of research studies

This is a case report. There is no requirement for registration.

## Guarantor

Takahiro Niikura.

## Provenance and peer review

Not commissioned externally peer reviewed.
